# The efficacy and safety of acupoint catgut embedding for peripheral facial paralysis sequela: A protocol for systematic review and meta-analysis

**DOI:** 10.1097/MD.0000000000027769

**Published:** 2021-12-10

**Authors:** Jingyun Ji, Yuchen Liu, Weijie Wen, Fengyi Wang, Rundong Tang

**Affiliations:** a Clinical Medical School of Acupuncture, Moxibustion and Rehabilitation, Guangzhou University of Chinese Medicine, Guangzhou, Guangdong, China; b The Bao‘an District TCM Hospital, The Seventh Affiliated Hospital of Guangzhou University of Chinese Medicine, Guangzhou University of Chinese Medicine, Shenzhen, Guangdong, China.

**Keywords:** acupoint catgut embedding, meta-analysis, peripheral facial paralysis sequela, protocol, systematic review

## Abstract

**Background:**

Peripheral facial paralysis sequela (PFPS) is a group of sequence syndrome after the acute onset of peripheral facial paralysis. Nearly 70% of patients with peripheral facial paralysis recover completely, but nearly 30% of patients leave multiple sequelae, which have serious negative impacts on the physical and psychological health of patients. Without a high risk of side effect, acupoint catgut embedding (ACE), a common acupuncture therapy, is widely used to treat this disorder. And a number of studies have shown the efficacy of this therapy for PFPS. But in fact, the evidence of the overall effect of ACE in the treatment of PFPS is still insufficient. Therefore, the purpose of this study is to evaluate the efficiency and safety of ACE for PFPS.

**Methods:**

Two reviewers will collect randomized controlled trials (RCTs) on ACE for PFPS by searching the following databases, including The Cochrane Library, PubMed, Web of Science, EMBASE, China Biomedical Literature (CBM), China National Knowledge Infrastructure (CNKI), Chinese Scientific Journals Database (VIP), and Wanfang database, from their initiation to May 2021. The searching of publications will include English and Chinese without any restriction of countries and regions. Besides, 2 reviewers will independently include in studies that meet the inclusion criteria and extract data we need, then use Cochrane Collaboration's Risk of Bias Tool to assess their methodological quality. The efficacy and safety of ACE as a treatment for PFPS will be assessed according to the synthetic risk ratio (RR), odds ratio (OR), or weighted mean difference (WMD), standardized mean difference (SMD) with consistent 95% confidence intervals (95% CI). And the Review Manager 5.3 software will be adopted to conduct the statistical analysis.

**Results:**

The protocol for meta-analysis will systematically evaluate the efficacy and safety of ACE for PFPS. And the final result of this search will provide sufficient evidence and an authentic assessment focusing on the problem.

**Conclusion:**

This search will explore whether ACE could be used as an effective and non-drug external therapy of TCM for PFPS and offer supports for clinical practice.

**PROSPERO Registration Number:**

CRD42021240004

## 1 Introduction

Peripheral facial paralysis (PFP), known as acute peripheral facial neuropathy, is caused by a partial or complete dysfunction in one side of the facial nerve.^[1]^ It is a commonly occurring disorder that mainly distorts the face image.^[2]^ The cardinal symptoms of PFP include unilateral muscle palsy, crooked mouth angle to the healthy side, obliteration of the naso-labial fold, facial muscle weakness on the affected side, dry eye, incomplete eyelid closure, headache, oral dysfunction during eating, dysphonia, the disappearance of forehead lines and pain behind the ear.^[3]^ It has been estimated that about 37 per 100,000 people suffer from PFP every year, which is reported in the relevant literature.^[4]^ The prevalence of males and females is almost the same.^[5,6]^ Besides, the prevalence also varies by age group, with people over the age of 70 more likely to get it.^[7]^ However, the underlying pathological factors and mechanisms are still poorly understood.^[8]^ Although there are many idiopathic cases, others are commonly associated with the causes, including herpes virus (herpes simplex virus or/and herpes zoster virus), cell-mediated immune response, otitis media, postsurgical complication, and local trauma of the facial nerve.^[9–11]^

Peripheral facial paralysis sequela (PFPS) is a group of sequence syndrome after the acute onset of PFP. Relevant studies have indicated that, because of abnormal regeneration of the facial nerve, nearly two-thirds of patients leave chronic and multiple sequelae such as incomplete eye closure, crocodile tears, oral dysfunction during eating and speaking, dysphonia, muscle contracture, facial joint movements, facial pain, and numbness.^[12,13]^ Thus, PFPS has a negative impact on the patient's quality of life and social function, both physically and psychologically.^[14]^ The common treatments of PFPS are corticosteroids, vitamin B, physiotherapy, and surgery. However, those treatments are difficult to obtain the obvious curative effect and with a high risk of side effects, including digestive and neurological symptoms, such as vomiting, nausea, diarrhea, dizziness, insomnia, and anxiety.^[11]^ In addition, the high cost of surgery is also out of reach for many families. Hence, there is an urgent need for an effective and convenient treatment to help patients relieve discomfort and promote recovery of facial function.

Increasingly, non-pharmaceutical therapies for PFP and its sequelae, including complementary and alternative medicine (CAM), may be selected because of the noxious side effects associated with pharmacological agents. Especially, CAM physiotherapies, such as yoga, massage, deep breathing, and meditation, were most frequently used by adults for headaches and various other pains, as well as anxiety, insomnia, and other similar mental conditions in the US.^[15]^ In China, clinicians mostly adopt acupuncture to treat neurological disorders, such as stroke, facial paralysis, trigeminal neuralgia, and others.^[16–18]^ In addition, acupuncture is recommended by the World Health Organization (WHO) as an alternative and complementary therapy for PFP and for promoting its later recovery.^[19]^

Acupoint catgut embedding (ACE) is a special type of acupuncture mainly by inserting threads (e.g., catgut or polydioxanone) into subcutaneous tissue or muscles at specific acupoints to prolong therapeutic stimulation. Because of its long-lasting efficacy and easy operation, this external therapy has been widely utilized in various chronic diseases in some Asian countries.^[20]^ In operation, with 2 components (a guide needle and the threads), ACE is actually a combination of traditional acupuncture and modern technology.^[21]^ It involves inserting the thread into the acupoint by a guide needle and then taking the needle out, leaving the thread under the skin or in the muscle layer.^[22]^ The catgut used is soft and absorbable. Over time, the catgut slowly softens, breaks down, dissolves, and is absorbed in the body.^[23]^ A number of previous studies have suggested that the utilization of ACE alone or combined with other therapeutic methods is effective for PFPS by repairing nerves and boosting immunity. But some of the results published appear to contradict each other. In fact, the evidence of the overall effect of ACE in the treatment of PFPS is still insufficient. Therefore, the purpose of this study is to comprehensively evaluate the efficiency and safety of ACE for patients with PFPS compared with other common therapies.

## 2 Methods and analysis

### 2.1 Study registration

This protocol has been registered on the International Prospective Register of Systematic Reviews (PROSPERO). Its PROSPERO registration number is CRD42021240004, which can be checked on this website (https://www.crd.york.ac.uk/PROSPERO/). Following the Preferred Reporting Items for Systematic Reviews and Meta-Analyses Protocol (PRISMA-P) statement guidelines, we will strictly conduct this study. And we have used and finished the PRISMA-P checklist when writing our report.^[24]^ Its full details are given in Appendix 1.

### 2.2 Eligibility criteria

#### 2.2.1 Type of studies

All the randomized controlled clinical trials (RCTs) on using ACE alone or combined with other therapies for PFPS will be included. The reports which are republished or lack of relevant outcome indicators or whose data cannot be extracted will be excluded. And the original studies whose diagnostic criteria did not meet with the clinical diagnosis of PFPS will also be eliminated. Moreover, the included studies must be published in English or Chinese.

#### 2.2.2 Type of participants

The studies included must be randomized controlled clinical trials. Participants were diagnosed with PFPS by clinical doctors according to the diagnostic criteria in the original study. The patients were not restricted by age, sex, and source. But those with other severe acute or chronic diseases must be excluded.

#### 2.2.3 Type of intervention

The included studies will be observed the effects of ACE. ACE means implanting lines (catgut sutures, protein sutures, absorbable sutures) under the skin of acupoints and continuously stimulating the meridians and acupoints. Patients in the treatment group will be given ACE whether or not combined with treatments received in the control group (acupuncture, electro-acupuncture, moxibustion, cortical hormone, a traditional Chinese herb, and cupping therapy, etc). There will be no special requirements for manipulation, frequency, acupuncture points, and duration. The control group could be treated with various other therapies but without ACE.

#### 2.2.4 Type of comparators

Patients in the control group will be given treatments (including acupuncture, electro-acupuncture, moxibustion, cortical hormone, traditional Chinese herb, and cupping therapy, etc) compared with the ACE treatment group.

#### 2.2.5 Type of outcome measures

##### 2.2.5.1 Primary outcomes

Effective rate; Scores of House-Brackmann facial nerve grading system.

##### 2.2.5.2 Secondary outcomes

Scores of Sunnybrook facial nerve grading system; Portmann score; Facial nerve conduction velocity (NCV); Incidence of adverse events.

### 2.3 Search strategy

We will collect RCTs on ACE for PFPS by searching the following databases: The Cochrane Library, PubMed, Web of Science, EMBASE, CBM, CNKI, VIP, and Wanfang database, from their initiation to May 2021. The searching of publications will include English and Chinese without any restriction of countries and regions. The search strategy will mainly be composed of keyword and Medical Subject Headings (MeSH) to seek the target reports, such as “acupoint catgut embedding” or “thread embedding acupuncture” or “acupoint thread embedding” and “randomized controlled clinical trails” and “peripheral facial paralysis” or “facial palsy” or “idiopathic facial paralysis” and “sequela” or “sequelae”, etc. Taking PubMed as an instance, the search strategy is summarized in Table 1, and it will be adjusted according to the search rules of different databases.

**Table 1:** The search strategy for PubMed.

Number	Search terms
#1	“facial paralysis”[MeSH Terms]
#2	“paralysis, facial”[Title/Abstract] OR “paralyses, facial”[Title/Abstract] OR “facial palsy”[Title/Abstract] OR “palsy, facial”[Title/Abstract] OR “hemifacial paralysis”[Title/Abstract] OR “paralysis, hemifacial”[Title/Abstract] OR “facial paresis”[Title/Abstract] OR “paresis, facial”[Title/Abstract] OR “facial paralysis, peripheral”[Title/Abstract] OR “facial paralyses, peripheral”[Title/Abstract] OR “peripheral facial paralysis”[Title/Abstract] OR “lower motor neuron facial palsy”[Title/Abstract] OR “upper motor neuron facial palsy”[Title/Abstract]
#3	“idiopathic facial palsy"[Title/Abstract] OR “facial neuritis”[Title/Abstract]
#4	#1 OR #2 OR #3
#5	“sequela”[Title/Abstract] OR "sequelae”[Title/Abstract]
#6	“acupoint catgut embedding”[Title/Abstract] OR “acupoint thread embedding”[Title/Abstract] OR ”thread embedding acupuncture[Title/Abstract] OR “catgut embedding, acupoint”[Title/Abstract] OR ”thread embedding, acupoint”[Title/Abstract] OR “acupuncture, catgut embedding”[Title/Abstract] OR ”acupuncture, thread embedding”[Title/Abstract]
#7	“randomized controlled trails”[MeSH Terms] OR "controlled clinical trail”[MeSH Terms]
#8	“randomized controlled trails”[Title/Abstract] OR ”controlled clinical trial”[Title/Abstract] OR “randomized”[Title/Abstract] OR ”randomised”[Title/Abstract]
#9	#7 OR #8
#10	#4 OR #5 OR #6 OR #9

### 2.4 Data collection and analysis

#### 2.4.1 Selection of studies

**Figure 1:**
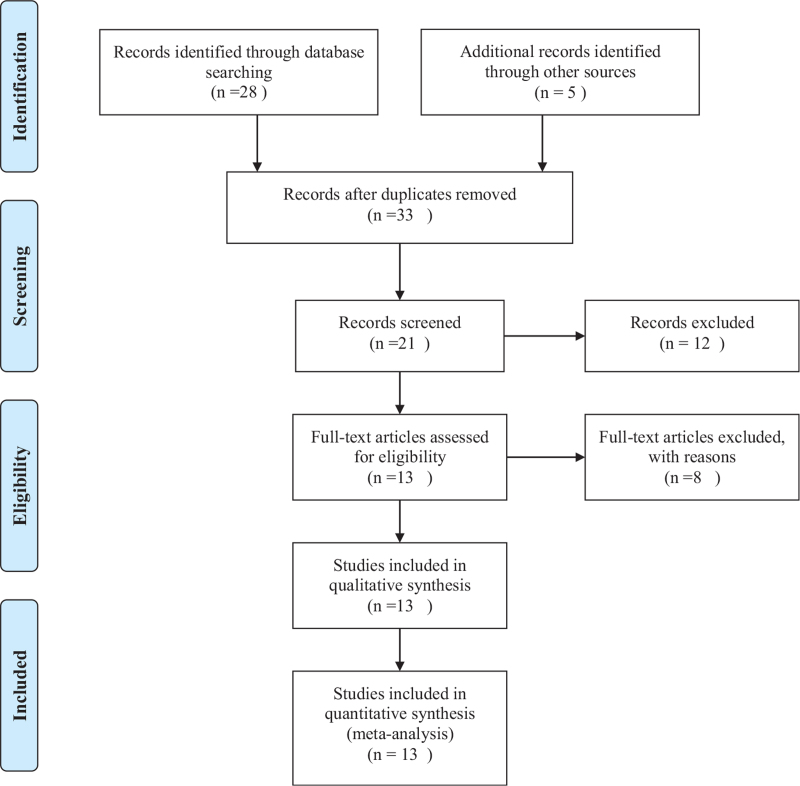
Flow diagram of study selection process.

We will retrieve all the literature needed from the databases according to correct subject terms and keywords, then eliminate the duplicate documents. Next, 2 reviewers will read the title and abstract of the literature independently to delete those not related to this systematic review. And the 2 reviewers will download the remaining documents to read their full text and respectively determine the final articles that meet the inclusion criteria. Any disagreement regarding selecting studies will be resolved by discussion or consultation with the third reviewer. The flow diagram of the selection process of included studies is shown in Fig. 1.

#### 2.4.2 Data extraction and management

The information extracted by the 2 review team members will be inputted into Excel 2010, which includes author, title, journal, year of publication, the country where the study was conducted, sample size, intervention measures, study methodology, outcome measurements, indicators of acceptability to users, and information for assessing the risk of bias. Two reviewers will extract data independently; discrepancies will be identified and resolved through discussion (with a third reviewer if necessary). We will request the missing data from the study authors.

#### 2.4.3 Risk of bias assessment

Two reviewers will independently assess the risk of bias in included studies by considering the following characteristics: randomization sequence generation, treatment allocation concealment, blinding method of participants or/and personnel, blinding method of outcome assessment, completeness of outcome data, selective outcome reporting, and other sources of bias. Besides, the Cochrane Collaboration's Risk of Bias Tool^[25]^ will be used to assess the methodological quality of every study included. The above 7 items will be respectively evaluated by 3 grades (“high,” “unclear,” and “low”). If there is any dissidence, the 2 reviewers can consult a third independent author.

#### 2.4.4 Measures of treatment effect

We will use Review Manager 5.3 software to carry out the data synthesis if the included studies are sufficiently homogeneous. Weighted mean difference (WMD) or standardized mean difference (SMD) will be used for continuous data. Risk ratio (RR) or odds ratio (OR) will be used for the analysis of dichotomous data. We will give a 95% confidence interval (95% CI) to both of the 2 types of data. If the quantitative synthesis is not appropriate, we will give up meta-analysis and use a general statistical description for the summary.

#### 2.4.5 Assessment of heterogeneity

The chi-square test will be used to calculate the heterogeneity of data. The heterogeneity degree depends on the value of *I*^*2*^, which reflects the proportion of the total variation in the effect size. If *I*^*2*^≤50%, the data state no heterogeneity, then it should be analyzed with the fixed-effect model. In contrast, if *I*^*2*^>50%, it shows obvious heterogeneity and needs to be analyzed by the random-effect model. Otherwise, the sources of heterogeneity will be further explored through sensitivity analysis and subgroup analysis.

#### 2.4.6 Assessment of publication bias

When more than 10 articles are included finally, we will evaluate the reporting biases by the funnel plot, which is constructed by effect size and standard error. If the funnel plot is symmetrical on both sides, it indicates that there is no obvious publication bias. If not, it suggests there are some biases. And quantitative assessment of reporting bias can be performed by the Egger test or Begg test.^[26,27]^

#### 2.4.7 Subgroup analysis

If there is obvious heterogeneity, we will dissect the causes of heterogeneity. Some subgroup analyses will be designed and performed. The data should be grouped according to the different causes, such as specific therapies combined with ACE in treatment groups (acupuncture, electro-acupuncture, moxibustion, cortical hormone, traditional Chinese herb and cupping therapy, etc), different treatment duration (1 month, 2 months, and 3 months), different treatment frequency (once a week, once 2 weeks, and once a month), different material of ACE (catgut suture, protein suture and absorbable suture), and the like. Then, the chi-square test will be adopted for each subgroup to observe the heterogeneity of the data. On the basis of their heterogeneity, a fixed-effect model or random-effect model will be utilized to perform the meta-analysis.

#### 2.4.8 Sensitivity analysis

Sensitivity analyses will be conducted to examine the robustness of heterogeneity. We will select the elimination method to observe the heterogeneity changes after each article is removed one by one. If the heterogeneity degree of the remaining articles decreases significantly after 1 article is excluded, it indicates that the source of heterogeneity is from the article. Further, in this article, we will explore the specific source from multiple aspects, such as experimental design, sample size, outcome indicators, evaluation criteria, etc.

#### 2.4.9 Grading the quality of evidence

We will select Grading of Recommendations Assessment, Development and Evaluation (GRADE) to evaluate the quality of evidence in each study included. The evaluation will be performed through the following 5 aspects: risk of bias, indirectness, inconsistency, imprecision, and publication bias.^[28]^

### 2.5 Ethics and dissemination

This study does not involve personal information and human trial data; therefore, it is not necessary to require an ethical approval. We intend to publish the final results of this study in a peer-reviewed scientific journal, as well as at some professional conferences.

## 3 Discussion

The characteristic findings of PFP are sudden onset of unilateral facial motor neuron injury, which leads to paralysis of involved facial muscles as well as reaches its peak by 72 hours.^[14]^ PFPS usually develops from acute PFP to chronic sequelae in the later stage, mainly manifests in facial pain, dysfunction of the related 5 senses, and appearance changes. Due to the severe condition or poor physique of patients, PFPS appears more and more frequently after the attack of facial paralysis. It significantly reduces the life quality of patients and brings them great physical and mental stress.

When facial paralysis develops to the sequelae stage, patients have commonly tried a variety of different treatment measures, but which are difficult to achieve an obvious effect. For instance, in Western medicine, botulinum injection, facial nerve decompression, facial nerve anastomosis, and other therapies can be adopted to treat PFPS. However, it is still inevitable to leave some different degrees of legacies, and their potential risk is also high.^[29]^ The application of ACE as the treatment of PFPS is simple, effective, and side effect free. In reality, clinicians have paid more and more attention to this external physiotherapy. Nevertheless, currently, ACE still lacks sufficient systemic and scientific evaluation of its efficiency and safety for PFPS. Therefore, this research aims to provide real and reliable evidence on that theme and promote the utilization of ACE for PFPS. Of course, there may be still some shortcomings in this review, such as the lack of RCTs with large samples and high quality, the failure to gain complete data from some articles and their authors, the collection without covering all relevant literature worldwide owing to language restrictions. In the future, we will make further improvements and deepening of this study according to the deficiencies mentioned above and the actual changes.

## Author contributions

**Conceptualization:** Jingyun Ji

**Data curation:** Jingyun Ji, Yuchen Liu

**Formal analysis:** Jingyun Ji, Yuchen Liu, Weijie Wen

**Funding acquisition:** Rundong Tang

**Guarantor of the review:** Jingyun Ji

**Investigation:** Fengyi Wang.

**Methodology:** Rundong Tang, Jingyun Ji

**Project administration:** Rundong Tang

**Software:** Jingyun Ji, Weijie Wen

**Validation:** Rundong Tang

**Visualization:** Fengyi Wang

**Writing – original draft:** Jingyun Ji, Yuchen Liu

**Writing – review and editing:** Rundong Tang, Weijie Wen
